# 1667. The Accuracy of Hospital-Onset Bacteremia and Candidemia Metric Variants Across Hospitals

**DOI:** 10.1093/ofid/ofac492.133

**Published:** 2022-12-15

**Authors:** Julia Lewis, Vanessa W Stevens, Brennan Ochoa, Colin Malone, Makoto M Jones

**Affiliations:** Salt Lake City VA Medical Center, Salt Lake City, Utah; US Department of Veterans Affairs, Salt Lake City, Utah; University of Utah, Denver, Colorado; US Dept of Veterans Affairs, Salt Lake City, Utah; University of Utah, Denver, Colorado

## Abstract

**Background:**

Hospital-onset bloodstream (HOB) infection metrics are automatable alternatives to metrics based on chart review and may have comparable accuracy. Our group explored and evaluated variants of these measures.

**Methods:**

Multiple electronic rules for HOB infection were developed, ranging from simple to moderately complex (Table 1).

To assess diagnostic accuracy overall and its stability over time and between facilities, we gathered electronic health record data between October, 2015 and December, 2019. Acute care admissions where the first positive blood culture was greater than 3 days after admission were included. We block-randomized 10 facilities across regions (North Atlantic, Southeast, Midwest, Continental, and Pacific) and whether above and below the median number of operating acute care beds. We restricted analysis to facilities > 30 acute care beds. Four reviewers reviewed approximately 50 charts randomly selected from the 10 facilities, with 10 charts overlapping with 2 other reviewers. A 5th reviewer, adjudicated in the case of disagreement or uncertainty. Accuracy statistics were generated by comparing algorithm outputs to the adjudicated standard.

**Results:**

Compared to the 140 chart standard, sensitivity was low for all algorithms investigated (Table 2), except where common skin commensals were excluded. Specificity dropped when antimicrobial-based criteria were used. Using definition F from Table 1 as representative of consistency of accuracy across facilities, the median facility sensitivity and specificity were 80.1% [IQR 72.1-95] and 85.4% [73.3-100], respectively.

**Conclusion:**

HOB metrics may need to exclude common skin commensals for simplicity and accuracy. Addition of antimicrobial use criteria did not seem to improve accuracy. Although sample sizes were small, accuracy metrics appeared relatively stable across facilities.

**Disclosures:**

**All Authors**: No reported disclosures.

